# Enhanced Adsorption of Trace Ethylene on Ag/NZ5 Modified with Ammonia: Hierarchical Structure and Metal Dispersion Effects

**DOI:** 10.3390/molecules29050981

**Published:** 2024-02-23

**Authors:** Ying Qi, Huaming Yang, Chunli Li, Hao Li

**Affiliations:** National-Local Joint Engineering Laboratory for Energy Conservation in Chemical Process Integration and Resources Utilization, School of Chemical Engineering and Technology, Hebei University of Technology, Tianjin 300130, China; 201911501005@stu.hebut.edu.cn (Y.Q.); 202211501018@stu.hebut.edu.cn (H.Y.)

**Keywords:** ethylene adsorption, silver loaded ZSM-5, ammonia modification, hierarchical porous structure, silver dispersion

## Abstract

Trace ethylene poses a significant challenge during the storage and transportation of agricultural products, causing over-ripening, reducing shelf life, and leading to food waste. Zeolite-supported silver adsorbents show promise for efficiently removing trace ethylene. Herein, hierarchical Ag/NZ5(X) adsorbents were prepared via different ammonia modifications, which featured enhanced ethylene adsorption ability. Ag/NZ5(2.5) exhibited the largest capacity and achieved near-complete removal at room temperature with prolonged efficacy. Characterization results indicated that the ammonia modification led to the formation of a hierarchical structure in the zeolite framework, reducing diffusion resistance and increasing the accessibility of the active sites. Additionally, desilication effects increased the defectiveness, generating a stronger metal–support interaction and resulting in a higher metal dispersion rate. These findings provide valuable insights into the development of efficient adsorbents for removing trace ethylene, thereby reducing food waste and extending the shelf life of agricultural products.

## 1. Introduction

Ethylene (C_2_H_4_), a ubiquitous plant hormone, plays a pivotal role in regulating plant growth and development. Remarkably, even at exceedingly low concentrations—five parts per billion (ppb), for instance—ethylene remains significantly effective for plants [1]. Specifically, the presence of trace amounts of ethylene can accelerate the ripening process in the storage and transportation of fruit and vegetables (F&V), leading to detrimental consequences and subsequent losses in commercial value [2]. Therefore, it is of paramount importance to develop effective strategies for removing trace ethylene from the storage and transportation environment of post-harvest products [3].

Adsorption stands out as an excellent method for ethylene removal, offering advantages such as cost effectiveness, high efficiency, and ease of operation. Generally, adsorption can be divided into physical adsorption and chemical adsorption, depending on the acting forces involved [4]. In fact, adsorbents have been being widely employed in F&V preservation for over a century. For instance, active carbon was already used as a physical adsorbent for the preservation of F&V in the 1940s [5]. Furthermore, numerous commercial ethylene scavengers based on adsorption, usually derived from porous materials such as zeolite [6,7], clay, and metal–organic frameworks [8,9], have been developed.

The van der Waals force, a relatively weak interaction compared to the chemical bonds or interactions formed during chemical adsorption, is the acting force behind physical adsorption. Consequently, the desorption of physically adsorbed ethylene could occur easily even with a slight increase in temperature [10]. Additionally, the ethylene concentration in the storage and transportation environment of F&V is generally below 100 ppm, which can be regarded as trace ethylene. Compared to chemical adsorption, the poor selectivity and low capacity make it difficult to remove trace ethylene by means of physical adsorption [11]. As a result, adsorbents that are driven by chemisorption are more suitable for removing ethylene to preserve F&V.

Zeolite, a common porous material, is widely used in industry but seldom employed as an ethylene scavenger due to its inability to adsorb trace ethylene [12]. However, the ethylene adsorption capacity of zeolite could be dramatically enhanced after doping with specific metal species. Corma et al. prepared a flexible pure silica zeolite (ITQ-55) for separating ethylene from ethane. Benefiting from its special pore topology and framework flexibility, ITQ-55 exhibited unprecedented selectivity of ~100 [13]. In addition, Farrusseng et al. developed a series of silver-exchanged zeolite A adsorbents for the separation of ethylene/ethane mixtures, which exhibited excellent performance over AgX adsorbents. These AgA adsorbents performed absolute molecular sieving separation, which was achieved through its pore size falling between the kinetic diameters of the two gases [14]. Similarly, Pereyra et al. prepared two AgA-Zeolite adsorbents via the partial replacement of Na^+^ in NaA with Ag^+^ by ion exchange, followed by calcination at different temperatures. Silver cations and reoxidizing silver species were identified as key factors for the high adsorption capacity and affinity towards ethylene [15]. Similar conclusions were drawn in a study on the selective separation of ethylene/ethane, which confirmed that separation performance is related to stable monovalent silver ions [16]. Very recently, we employed Ag/ZSM-5(130) as an adsorbent for trace ethylene removal, which achieved a capacity of 4.89 mL·g_ads_^−1^ at room temperature, primarily thanks to the π-complexation of Ag^+^ in the adsorbent [17].

In fact, the adsorption capacity of transition metal-loaded adsorbents is determined by two aspects: the active metal species and the properties of the supports [18]. The increase in the loading content of the active metal species can improve the adsorption performance to some extent but simultaneously leads to increased cost, which limits its application in larger scales [19]. Alternatively, the modification of the supports by acidic or alkaline solutions to change their physical/chemical properties proved to be another effective way for improving the adsorption performance [20].

Aqueous ammonia is commonly used as a modification agent of heterogeneous catalysts with a variety of advantages, including low toxicity, mild alkalinity, no residue after calcination, low cost, etc. Zhao and Ma et al. prepared a Ni/SiO_2_ catalyst for syngas methanation by ammonia-assisted impregnation [21]. This catalyst showed good activity due to high nickel dispersion and strong metal–support interaction compared to the catalyst prepared by traditional impregnation. Verboekend and Sels et al. designed a hierarchical USY catalyst via a post-synthetic treatment with ammonia [22]. The treatment with aqueous ammonia of USY resulted in the formation of mesopores, leading to excellent performance of the modified USY in the acid-catalyzed isomerization of α-pinene and the metal-catalyzed conjugation of safflower oil. Moreover, Janiszewska and Kowalska-Kuś et al. investigated the post-synthetic modification of a silicalite-1 (MFI) acetalization catalyst with ammonium solutions. The partial removal of external silanol groups and the formation of acidic internal isolated as well as hydrogen-bonded OH groups afforded the modified silicalite-1 with a high catalytic activity for the acetalization of glycerol with acetone and high selectivity towards 2,2-dimethyl-1,3-dioxolane-4-methanol [23]. The selective adsorption of ethylene on transition metal-doped adsorbents shares some foundational principles with heterogeneous catalysis. Therefore, the modification of catalysts with ammonia might be expanded to the development of advanced materials for the selective adsorption of ethylene and finally applied to the removal of trace ethylene in the storage and transportation of F&V.

Herein, a silver-doped HZSM-5 zeolite is modified with different amounts of aqueous ammonia, followed by calcination to afford modified adsorbents that show enhanced ethylene adsorption. The investigation results reveal that the modification introduces mesopores into the adsorbents, forming a hierarchical pore structure, which decreases the diffusion resistance and increases the accessibility of the active sites. In addition, the desilication effect of ammonia creates more defective sites on the surface, resulting in a stronger metal–support interaction, which then increases the metal dispersion rate and ultimately enhances the adsorption of ethylene. These findings illustrate a promising route for improving the performance of zeolite-supported ethylene adsorbents, which has big application prospects in the field of F&V preservation.

## 2. Results and Discussion

### 2.1. Structure and Surface Morphology of Ag/NZ5(X)

**Figure 1:**
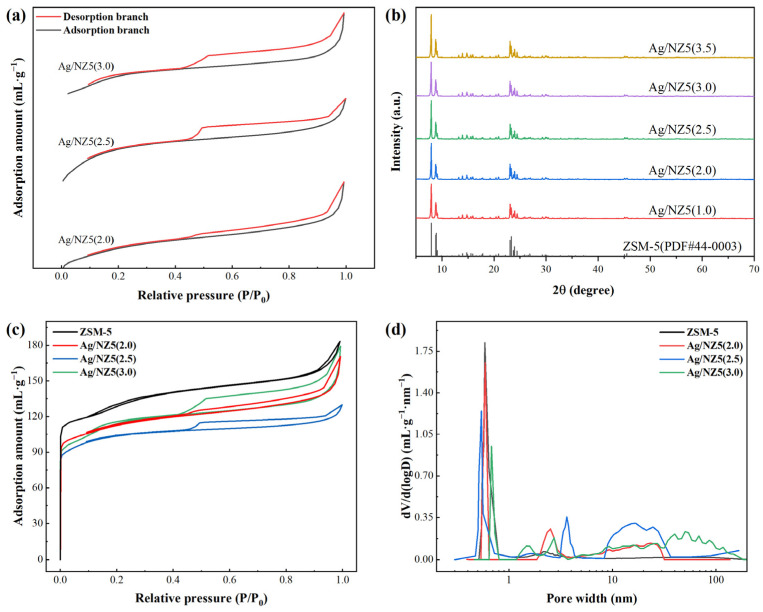
(**a**) Enlarged N_2_ adsorption/desorption isotherms of Ag/NZ5(X) and unmodified ZSM-5, indicating pore volume and surface area variations. (**b**) XRD patterns, revealing structural changes across different ammonia modifications. (**c**) Complete N_2_ adsorption/desorption isotherms, providing comprehensive insights. (**d**) Pore size distributions of the samples, obtained by adopting NLDFT.

The ammonia etching effect on the zeolite during adsorbent preparation has the potential to alter the pore structure of Ag/NZ5(X), thereby impacting its ethylene adsorption performance. Therefore, the pore structure of Ag/NZ5(2.0), Ag/NZ5(2.5), Ag/NZ5(3.0), and parent zeolite (i.e., unmodified ZSM-5 zeolite) were analyzed by N_2_ adsorption–desorption isotherms. As shown in Figure 1a, all the samples show type II-like isotherms with an H4 type hysteresis loop (red curves) according to IUPAC classification, indicative of mesoporous structures within the Ag/NZ5(X) samples [24]. Notably, the significant N_2_ uptake at low relative pressures in Figure 1c also suggests the preservation of microporosity, likely due to the high SiO_2_/Al_2_O_3_ ratio of the initial HZSM-5 material [25]. In addition, the pore size distributions of the samples in Figure 1d further corroborate the aforementioned description. These results suggest that mesopores are introduced into the zeolite structure through desilication during the ammonia modification process, typically at the expense of micropores.

To quantitatively assess the impact of this modification, the Indexed Hierarchy Factor (IHF) is employed. The IHF provides a normalized comparison of the microporous and mesoporous volumes to their respective maximum values [26]. Consequently, the IHF was calculated from the N_2_ adsorption–desorption isotherms, as outlined in the literature, to assess the pore structure alterations in Ag/NZ5(X). The IHFs, textural properties, and the method used for calculating the IHF are presented in Table 1.

As shown in Table 1, the total specific surface areas of Ag/NZ5(X) samples changed slightly and randomly with an increase in ammonia volume during modification, while the mesopore specific surface areas increased, indicating the successful introduction of mesopore structures. Additionally, the micropore volume decreased with the increase in ammonia volume. The results suggest sufficiently that the modification of Ag/NZ5(X) samples with ammonia leads to successful conversion of micropores into mesopores, consistent with the mechanisms reported in prior studies [27]. The IHF is thus employed to quantify the mesopore development per the corresponding decrease in micropore volume during the ammonia modification process. The IHF of Ag/NZ5(X) initially rises with an increase in ammonia volume, reaching a peak with Ag/NZ5(2.0) at an IHF of 0.68, before subsequently declining (Table 1). The results suggest that an optimal ammonia level exists where micropores are efficiently transformed into mesopores. Beyond this optimal point, the etching effect of ammonia intensifies with higher concentrations, which creates larger mesopores and paradoxically leads to a decrease in the number of mesopores originated from micropores [28]. Therefore, the careful modulation of ammonia during modification is pivotal in establishing a hierarchical pore structure. Such a structure is likely to facilitate contact between ethylene molecules and the active sites, ultimately improving the adsorption efficacy and increasing the utilization rate of Ag/NZ5(X).

As depicted in Figure 1b, the XRD patterns of Ag/NZ5(X) samples retain the characteristic peaks of ZSM-5 zeolite across all samples, indicating no significant changes in the framework structure, confirming the structural integrity of the adsorbents post modification [29]. Subtle variations in peak intensities suggest a reduction in relative crystallinity (calculated from the peaks located at 2θ = 23–25°), which is consistent with the desilication effect attributed to ammonia treatment [30]. Moreover, the XRD patterns show no visible silver diffraction peaks, which implies that the average size of silver nanoparticles (NPs) is beyond the range of XRD detection (4–5 nm), indicating that the silver NPs formed on the adsorbents are small and uniformly dispersed [31].

**Figure 2:**
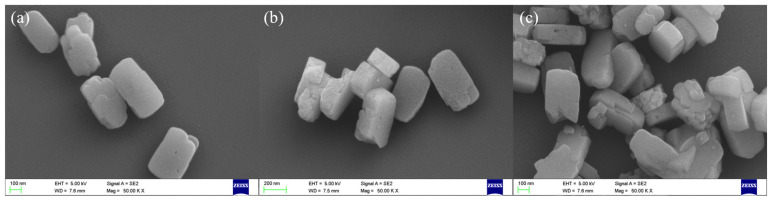
Scanning electron microscopy (SEM) images of Ag/NZ5(X) samples post modification, showing the preserved coffin-like morphology characteristic of ZSM-5: (**a**) Ag/NZ5(2.0) with the smoothest surface among the samples; (**b**) Ag/NZ5(2.5); (**c**) Ag/NZ5(3.0).

The impact of ammonia modification on the surface morphology of Ag/NZ5(X) samples was examined using electron microscopy. Figure 2 displays SEM images of three Ag/NZ5(X) samples, revealing that the characteristic coffin-like morphology of ZSM-5 zeolite is preserved post modification. Notably, the surface of Ag/NZ5(2.0) appears smooth compared to the others, without any significant irregularities.

Ag/NZ5(2.5) exhibits an intense surface roughness compared to Ag/NZ5(2.0), accompanied by the emergence of pits that may represent pore structures. Additionally, the surface of Ag/NZ5(3.0) appears even rougher and more irregular. Notably, the relatively mild alkalinity of ammonia results in no significant impact on the overall morphology of Ag/NZ5(X). The observed changes in surface characteristics align with the regulated formation of mesopores, a phenomenon dependent on the amount of ammonia added during preparation, which is also consistent with the findings from N_2_ adsorption–desorption analysis.

**Figure 3:**
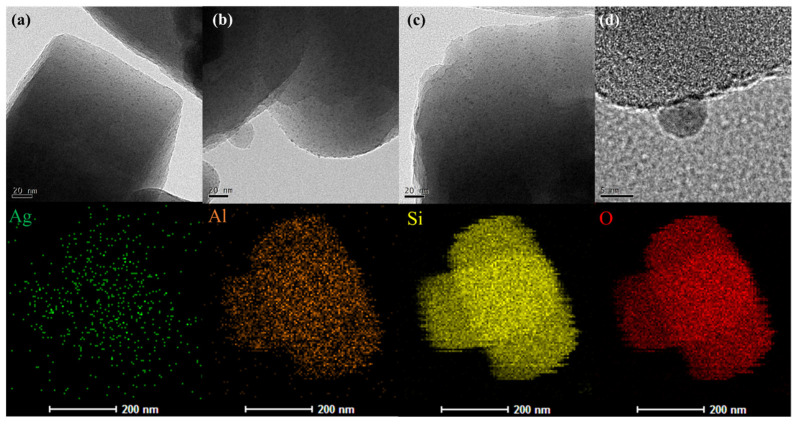
TEM images of Ag/NZ5(X) (**top**): (**a**) Ag/NZ5(2.0); (**b**,**d**) Ag/NZ5(2.5) with silver nanoparticle on the surface; (**c**) Ag/NZ5(3.0). Mapping analysis results of Ag/NZ5(2.5) (**bottom**), with the corresponding TEM image shown in Figure S2b.

The TEM images presented in Figure 3 distinctly reveal the deposition of silver NPs on the surface of Ag/NZ5(X) samples. According to Figure S2a, the average diameter of these silver NPs is 2.9 nm, which is in agreement with the above results. Moreover, the wide pore size distribution of Ag/NZ5(X) allows the silver NPs to reside in the pores, facilitating the uniform dispersion of these silver NPs. The uniform dispersion of silver NPs then establishes effective adsorption sites for ethylene [32]. It is worth mentioning that the specific surface area and micropore volume of all the Ag/NZ5(X) samples is lower than ZSM-5, which could be ascribed to the silver NPs entering the pore of zeolite and partially blocking it. Moreover, the lattice fringes of the supporter ZSM-5 in the images remain clear. This indicates that the crystal structure of ZSM-5 remains mostly intact during the modification, which is in line with the characterization results of XRD [33]. Consequently, it can be inferred that the etching effect of ammonia on the adsorbents is relatively mild, and its deterioration on the crystallinity of ZSM-5 is limited.

In the high-resolution TEM image (Figure 3d), lattice fringes are visible on the silver NPs, indicating tight bonding between the silver NPs and zeolite [34]. Furthermore, the element mapping analysis results in Figure 3 confirm a relatively uniform distribution of the Ag element without evident agglomeration, also consistent with the XRD results. Thus, the conclusion can be drawn that the ammonia modification does not induce the agglomeration of active components in Ag/NZ5(X).

Based on the aforementioned results, we can conclude that the zeolite micropores can be effectively transformed into mesopores via ammonia modification, establishing a hierarchical structure in Ag/NZ5(X). This structural modification is expected to strengthen ethylene adsorption without significantly impacting the crystal structure of the zeolite-based adsorbents. Optimal modification results in adsorbents with a higher IHF value, indicating an enhanced hierarchical degree, which leads to reduction in diffusion resistance, thus improving the accessibility of ethylene molecules to active sites, ultimately enhancing the adsorption performance. Additionally, the uniform dispersion of silver NPs on the adsorbent surface without evident agglomeration further contributes to effective ethylene adsorption.

### 2.2. Defectiveness and Metal Dispersion of Ag/NZ5(X)

**Figure 4:**
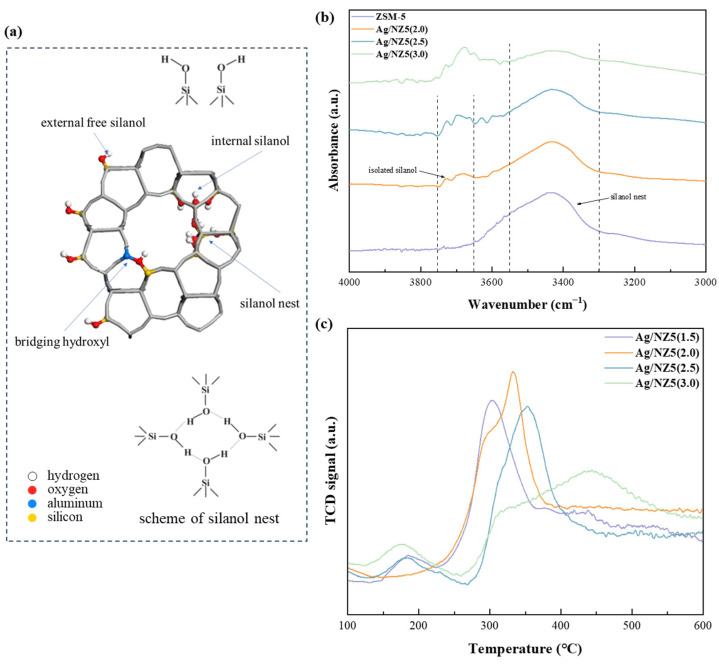
(**a**) Diagrammatic representation of silanol and hydroxyl at various positions; (**b**) FT-IR spectra of Ag/NZ5(X) and ZSM-5 in the OH stretching vibrations range, identifying the existence of different species; (**c**) H_2_-TPR profiles of Ag/NZ5(X), revealing the intensity of the metal–support interaction.

The Ag/NZ5(X) adsorbents were derived from the doping of silver species into the matrixes of ammonia-modified HZSM-5. There are different types of silanol groups in HZSM-5 microporous zeolites, including external and internal Si-OH and nest Si-OH, together with stronger bridging Si-OH-Al Brønsted sites, as shown in Figure 4a. The absorption peak observed in the Fourier transform infrared spectroscopy (FT-IR) spectrum, ranging between 3000–4000 cm^−1^, is attributed to the stretching vibrations of these silanol groups, providing valuable insights into zeolite’s defective sites [35]. The characteristic stretching frequency of OH groups decreases due to the formation of hydrogen bonds with other species, leading to distinct FT-IR peaks corresponding to different defective structures (Figure 4a) [36]. For instance, the broad symmetric band within 3300–3550 cm^−1^ signifies the presence of silanol nests (i.e., Si-OH groups interconnected by hydrogen bonds) in the zeolite. Additionally, the absorption band at around 3600–3650 cm^−1^ corresponds to the bridging hydroxyl groups between silica and aluminum atoms in the zeolite framework, typically identified as Brønsted acid sites [37]. The bands between 3650 and 3750 cm^−1^ represent the isolated silanol groups. Within this range, the absorption peak at around 3690 cm^−1^ corresponds to internal silanol groups, while the peak at around 3720 cm^−1^ corresponds to external silanol groups [38]. Importantly, the intensity of these absorption peaks serves as an indicator of zeolite defectiveness, with higher intensity reflecting greater defectiveness [39].

During ammonia modification, prolonged exposure to aqueous ammonia leads to a gradual hydrolysis of the Si–O–Si bonds in the zeolite framework, resulting in a defective structure. This desilication effect primarily impacts silanol nests, causing a decrease in the intensity of their FT-IR absorption band [40,41]. Consequently, the silanol nest structure undergoes transformation, evolving into isolated silanol groups [42]. In Figure 4b, all the samples exhibit broad bands between 3300–3550 cm^−1^ representing silanol nests. Besides ZSM-5, all three Ag/NZ5(X) samples also exhibit two peaks corresponding to isolated silanol groups. As ammonia concentration increases, the intensity of silanol nests decreases, while the intensity of isolated silanol groups increases, consistent with the aforementioned desilication process.

Ag/NZ5(X) adsorbents, considered as a type of metal-loaded materials, exhibit performance sensitivity to both metal dispersion conditions and silver loading contents [43]. Previous studies highlight the Si/Al ratio as a critical factor influencing material properties and performance [44,45]. Despite the initial Si/Al ratio of ZSM-5 being 130 in this study, the Si/Al ratio of Ag/NZ5(X) samples may change with the treatment with different ammonia amounts, potentially impacting adsorption performance. To address this, silver loading contents, metal dispersion rates, and actual Si/Al ratios of Ag/NZ5(X) were systematically investigated, with results detailed in Table 2.

The silver loading contents and real Si/Al ratios across various Ag/NZ5(X) samples exhibit a consistent trend, showing slight variation with increasing ammonia amounts. The real Si/Al ratios in all four adsorbent samples decreased from the initial 130 to approximately 90, indicating a minor influence of ammonia concentrations on Si/Al ratio reduction, similar to the results in the literature [46]. During ammonia modification, Si and Al atoms were simultaneously extracted from the zeolite framework with a higher preference for Si. Extracted Al species then recombined with zeolite, forming extra-framework aluminum species, leading to a decreased Si/Al ratio [47]. Importantly, the modification process is significantly influenced by treatment time and temperature, the higher the temperature and the longer the time, the stronger the desilication effects. Additionally, the silver loading contents maintain a consistent value of around 0.5 wt.%, below the theoretical 1 wt.% (calculated from the AgNO_3_ amount used during the preparation of the adsorbents). These findings suggest that ammonia modification has no significant impact on silver loading contents and real Si/Al ratios in Ag/NZ5(X).

With an increasing amount of ammonia, the metal dispersion rate of Ag/NZ5(X) adsorbents exhibits a rising trend, reaching 23.32% for Ag/NZ5(3.0). The regulation of the metal dispersion rate by the ammonia amount during preparation is evident, attributed to the generation of defective sites through ammonia treatment. Generally, relatively small nanoparticles tend to spontaneously agglomerate to reduce the surface energy of the NPs, resulting in a poor metal dispersion rate. However, the presence of defective sites enhances the interaction between metal NPs and the support, acting as anchor points and boosting the metal dispersion rate [34,48]. These interactions were further characterized using hydrogen temperature-programmed reduction (H_2_-TPR), revealing three different reduction temperatures appear at around 180 °C, 330 °C, and 450 °C, as shown in Figure 4c.

The reduction temperature in H_2_-TPR profiles represents the intensity of the metal–support interaction in materials. Notably, the shifting of reduction peaks towards higher temperatures of Ag/NZ5(2.5) suggests a stronger metal–support interaction in Ag/NZ5(2.5) compared to that in Ag/NZ5(2.0) and Ag/NZ5(1.5). Furthermore, Ag/NZ5(3.0), with an increasing amount of ammonia in modification process, exhibits a reduction peak around 450 °C, indicating even stronger metal–support interactions than those in the other samples. The abundance of hydroxyl groups at defective sites plays a crucial role in the dispersion of metal NPs, facilitating interactions with active metal species through hydrogen bonding. This interaction serves as an anchoring point, contributing to the active metal uniformly dispersing on zeolite [21,49].

In summary, the defect concentration in the framework of Ag/NZ5(X) increases with the increase in ammonia amount during the modification process, leading to a more robust metal–support interaction and promoting the uniform dispersion of silver species. The highly dispersed active silver species could eventually enhance the performance of Ag/NZ5(X) in adsorbing trace ethylene.

### 2.3. Ethylene Adsorption Performance of Ag/NZ5(X)

**Figure 5:**
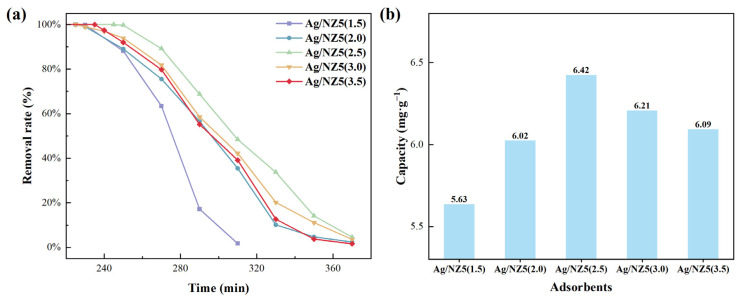
(**a**) Breakthrough curves of Ag/NZ5(X) illustrating the effective removal of ethylene. (**b**) Ethylene adsorption capacity, highlighting the superior performance of Ag/NZ5(2.5) with the largest capacity.

The Ag/NZ5(X) adsorbents were evaluated in the removal of trace ethylene from the air at room temperature. Notably, the ethylene removal capacity of Ag/NZ5(2.5) exhibits a decline with increasing temperature, affirming that the samples in this study eliminate ethylene primarily through adsorption rather than catalytic oxidation (Figure S3). The breakthrough curves for the adsorption of trace ethylene by the Ag/NZ5(X) adsorbents, illustrated using GC data from the experiments, are presented in Figure 5.

All the adsorbents except for Ag/NZ5(1.5) demonstrate a similar trend for removing ethylene within the test time of 6 h under the same experimental conditions as shown in Figure 5a. It is worth mentioning that the adsorption capacities of all the Ag/NZ5(X) samples surpass that of non-modified Ag/ZSM-5(130). Furthermore, all adsorbents display steep breakthrough curves, indicating effective ethylene removal even at lower concentrations [32]. These outcomes are attributed to the distinct hierarchical degrees and metal dispersion rates of Ag/NZ5(X) samples, regulated by the ammonia amount as described earlier. As can be seen from Figure 5b, the adsorption capacities of Ag/NZ5(X) follow an initial increase with the rise in ammonia amount used during the modification and reach the maximum at 2.5 mL of ammonia, followed by a subsequent decrease with a further increase in ammonia amount. The adsorption capacity ranking of Ag/NZ5(X) adsorbents is as follows: Ag/NZ5(1.5) < Ag/NZ5(2.0) < Ag/NZ5(3.5) < Ag/NZ5(3.0) < Ag/NZ5(2.5). Notably, Ag/NZ5(2.5) exhibits the best ethylene adsorption performance, maintaining near-complete removal for 255 min and achieving an adsorption capacity of 6.42 mg·g^−1^ at 25 °C.

The recycle test of Ag/NZ5(2.5) was conducted to validate its practicality. The loading amount of Ag/NZ5(2.5) was reduced from 500 mg to 200 mg for efficiency. After each run, the sample of Ag/NZ5(2.5) was calcined at 400 °C under a nitrogen atmosphere for 1 h. The regenerated sample of Ag/NZ5(2.5) was subjected to the next run under the same conditions. As shown in Figure S4, the adsorption capacity for ethylene was recovered over 93% in the second run, and with a retention close to 90% after six cycles. The decrease in adsorption capacity might be ascribed to the changes in silver valence and (or) leaking of active components [50].

The ethylene adsorption isotherms of Ag/NZ5(X), reflecting the strength of ethylene adsorption, can be fitted with isotherm equations to obtain useful parameters. Previous studies indicate the presence of both physical and chemical adsorption during the ethylene removal process [51]. The cation–π interaction and the interaction between ethylene CH groups and zeolite framework O elements drive the process, exhibiting bond energies comparable to hydrogen bonding [52]. Therefore, the Langmuir isotherm equation is not suitable for the process and needs some additional parameters as follows:

(1) $$q=\frac{q_{m,p}b_{p}p}{1+b_{p}p}+\frac{q_{m,c}}{2s}\ln\frac{1+b_{c}pe^{s}}{1+b_{c}pe^{-s}}\;$$

where *q_m_* is the saturated adsorption capacity; subscripts *p* and *c* represent physical and chemical adsorption; *b* is the Langmuir constant, which represents the strength of adsorption; *e* is the base of natural logarithms; *s* is a parameter related to the distribution of chemisorption energy, which reflects the uneven degree of the adsorption center on the adsorbent surface [53].

The ethylene adsorption isotherms of Ag/NZ5(2.0), Ag/NZ5(2.5), and Ag/NZ5(3.0) were measured at 25 °C. According to Figure S5b, all three samples demonstrate similar trends, with the amount of adsorbed ethylene significantly increasing as the ethylene partial pressure rises. Furthermore, the isotherms exhibit substantial curvature, consistent with the steep breakthrough curve in Figure 5a, indicating that the Ag/NZ5(X) adsorbents can efficiently adsorb ethylene even at lower concentrations [54].

The fitting results of the ethylene adsorption isotherms summarized in Table 3 demonstrate high satisfaction, with correlation coefficients close to 1. At higher partial pressures of ethylene, physical adsorption prevails, shifting toward an increased proportion of chemisorption as the ethylene partial pressure decreases. For Ag/NZ5(2.5), the chemically adsorbed ethylene molecules were 3.78 times higher than the physically adsorbed ones when the ethylene partial pressure dropped to 1 kPa. This suggests that the trace ethylene removal ability of Ag/NZ5(X) primarily arises from chemisorption. Additionally, the obtained b_c_ values from the fitting results are significantly larger than the b_p_ values, further indicating the dominant role of chemisorption in adsorbing trace ethylene by the Ag/NZ5(X) adsorbents.

**Figure 6:**
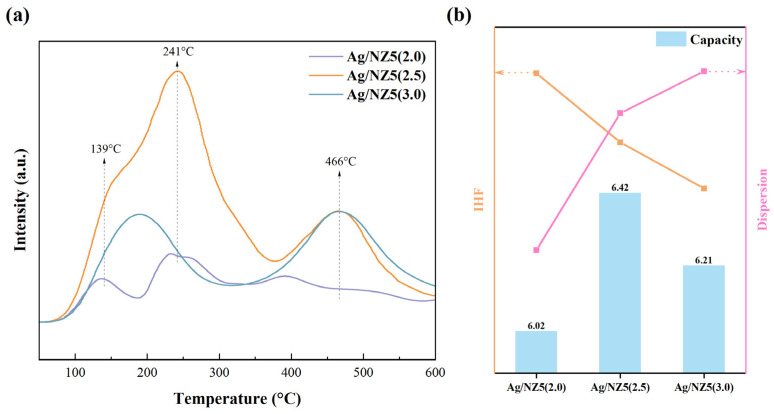
(**a**) Ethylene temperature-programmed desorption (TPD) profiles of Ag/NZ5, revealing different adsorption sites. (**b**) Correlation between capacity, Indexed Hierarchy Factor (IHF), and dispersion of silver species, providing insights into adsorption process.

To investigate the strength and distribution of adsorption sites in Ag/NZ5(X), temperature-programmed desorption of ethylene (C_2_H_4_-TPD) was conducted, and the results are presented in Figure 6a. Based on the ethylene desorption temperatures, three distinct adsorption site types were identified in Ag/NZ5, categorized as physical and chemical adsorption sites. Specifically, the desorption peak near 139 °C corresponds to physical adsorption sites, while peaks near 241 °C and 466 °C signify weaker and stronger chemical adsorption sites, respectively [51]. Consequently, both Ag/NZ5(2.5) and Ag/NZ5(3.0) exhibit chemical adsorption sites with high strength, whereas Ag/NZ5(2.0) includes chemisorption sites with lower strength. These findings indicate the coexistence of ethylene adsorption sites with varying strengths in Ag/NZ5(X) adsorbents. Notably, chemical adsorption predominantly governs trace ethylene adsorption in these adsorbents, aligning with the conclusions drawn from the adsorption isotherms.

To demonstrate the potential of Ag/NZ5(X) for preserving F&V, a banana storage experiment was conducted employing Ag/NZ5(2.5) as ethylene adsorbents. Bananas imported from Myanmar and purchased from a local fruit wholesale market were utilized for the experiment. The experiment lasted for 25 days, and the results depicted in Figure S6 indicate a noticeable delay in ripening time by 3–4 days for bananas treated with Ag/NZ5(2.5). Furthermore, at the conclusion of the experiment, the bananas treated with Ag/NZ5(2.5) remained in an edible state, while those in the control group had completely softened and turned yellow, with their internal liquid having leaked into the sealed bags by the final day, rendering them inedible. These results validate the preservation efficacy of Ag/NZ5(X) on F&V, which could be attributed to its ability to adsorb trace ethylene from air.

Based on the comprehensive results of adsorption experiments and characterization, we conclude that ammonia modification results in the formation of a hierarchical structure and promotes the uniform dispersion of silver species in the Ag/NZ5(X) framework, enhancing the adsorption of trace ethylene. The structure–activity relationship reveals an inverse correlation between the amount of ammonia as a modification agent in the preparation of Ag/NZ5(X) adsorbents and hierarchical degree as well as metal dispersion (Figure 6b). Notably, Ag/NZ5(2.5) exhibits a superior hierarchical degree and metal dispersion, synergistically yielding optimal ethylene adsorption. In general, the ammonia modification of Ag/NZ5(X) enhances its adsorption performance, thus increasing trace ethylene removal efficiency.

## 3. Materials and Methods

### 3.1. Materials and Reagents

HZSM-5 zeolite with the SiO_2_/Al_2_O_3_ ratio of 130 was purchased from the Catalyst Plant of Nankai University (Tianjin, China); silver nitrate (AgNO_3_) was purchased from Beijing InnoChem Science & Technology Co., Ltd. (Beijing, China); aqueous ammonia (NH_3_·H_2_O) was purchased from Tianjin Fengchuan Chemical Reagent Technologies Co., Ltd. (Tianjin, China). All the materials and reagents were used as received without any purification.

### 3.2. Preparation of the Adsorbents

The ammonia-modified adsorbents were prepared by using a typical incipient wetness impregnation method. Initially, 1 wt.% of silver nitrate (ca. 31.5 mg) was dissolved in deionized water to yield a transparent solution, which was subsequently stored in the absence of light. To this solution, different volumes of aqueous ammonia (1.5, 2.0, 2.5, 3.0, and 3.5 mL) were meticulously added until the total volume reached 4.5 mL. Subsequently, 3 g of HZSM-5 zeolite was placed in a beaker, and the mixed solution was added to the zeolite dropwise. It should be noted that the addition of the solution must be maintained dropwise to ensure the reproducibility of the results. After ultrasonication for 30 min, the white mixture was dried under a vacuum environment overnight at 40 °C and then dried under atmospheric pressure for 120 min at 80 °C. The dried white solid obtained after drying was ground into white powder and subsequently calcined in a muffle furnace at 550 °C for 300 min, and the ramp rate for calcination was set to 3 °C/min. Finally, the resulting white powder was ground once more and labeled as Ag/NZ5(X), where *X* represents the volume of aqueous ammonia used during adsorbent preparation. All the Ag/NZ5 samples were stored in a dark and dry environment and used for characterization and evaluation without any further treatment.

### 3.3. Ethylene Adsorption Experiment

The evaluation of ethylene adsorption performance was conducted in a 10 mm fixed-bed reactor, as illustrated in Figure S1. In this setup, a mixture of 500 mg of Ag/NZ5(X) and 500 mg of silicon dioxide (used as physical supports) were packed in the reactor. The composition of the ethylene standard gas comprised 100 ppm of ethylene, 21 wt.% oxygen, and approximately 79 wt.% nitrogen.

Breakthrough curves were used to evaluate the ethylene adsorption capacity and the reusability of Ag/NZ5(X). For measuring the breakthrough curves, an Agilent 7820A gas chromatograph (GC) was used to determine the ethylene concentrations of the gas stream. The flow rate of the gas stream was kept at 85 mL·min^−1^ by using a mass flow controller (MFC) under the conditions of 25 °C and atmospheric pressure. Finally, the ethylene removal rate (R) and adsorption capacity (C) of Ag/NZ5(X) were calculated by using equations provided in the Supporting Information. The optimal performance among the samples is defined by the longest sustained near-complete ethylene removal (i.e., R > 99%) coupled with the highest adsorption capacity. This dual metric serves as the benchmark for determining the best ethylene adsorption performance in the study.

## 4. Conclusions

In summary, hierarchical Ag/NZ5(X) adsorbents with uniform silver dispersion were prepared through the introduction of ammonia during incipient wetness impregnation. The introduction of ammonia poses two significant impacts to the adsorbents. Firstly, it transforms some micropores into mesopores, creating a hierarchical structure in Ag/NZ5(X), which reduces diffusion resistance and thus enhances accessibility to active sites. Secondly, it increases the defectiveness of Ag/NZ5(X) via desilication, leading to a stronger metal–support interaction and then a higher metal dispersion rate. These impacts synergistically contribute to the enhanced trace ethylene adsorption performance of Ag/NZ5(X). Notably, Ag/NZ5(2.5) exhibited outstanding trace ethylene adsorption performance and good reusability, maintaining near-complete removal for 255 min and achieving an adsorption capacity of 6.42 mg·g^−1^ at 25 °C. It was revealed that the removal of ethylene is primarily achieved through a chemisorption process, even at low concentrations. Finally, we hope that this work can provide valuable insights into high-performance adsorbent preparation, offering potential applications in the preservation of F&V.

## Figures and Tables

**Table 1:** The textural properties and the indexed hierarchy factor (IHF) of Ag/NZ5(X) and ZSM-5.

Adsorbent	S_total_^a^	S_meso_^b^	V_micro_^b^	V_total_^c^	IHF ^d^
	(m^2^·g^−1^)	(m^2^·g^−1^)	(cm^3^·g^−1^)	(cm^3^·g^−1^)	
Ag/NZ5(1.5)	351	35	0.199	0.237	0.59
Ag/NZ5(2.0)	364	44	0.184	0.231	0.69
Ag/NZ5(2.5)	355	47	0.165	0.226	0.66
Ag/NZ5(3.0)	356	50	0.149	0.212	0.63
Ag/NZ5(3.5)	353	59	0.115	0.205	0.58
parent zeolite	377	19	0.233	0.253	-

^a^ Specific surface area obtained by using the BET equation (P/P_0_ = 0.04–0.32). ^b^ Mesopore specific surface area and micropore volume calculated by adopting the t-Plot method. ^c^ Total volume calculated at P/P_0_ = 0.99. ^d^ IHF = (S_meso_/59 m^2^·g^−1^) × (V_micro_/0.199 cm^3^·g^−1^).

**Table 2:** The silver loading contents, metal dispersion rate, and real Si/Al ratio of Ag/NZ5(X).

Adsorbent	Silver Loading ^a^ (wt.%)	Dispersion Rate ^b^ (%)	Si/Al Ratio ^a^
Ag/NZ5(1.5)	0.528	10.93	95
Ag/NZ5(2.0)	0.512	12.45	89
Ag/NZ5(2.5)	0.499	20.77	86
Ag/NZ5(3.0)	0.495	23.32	92

^a^ The silver loading contents and real Si/Al ratio were obtained by using ICP characterization. ^b^ The metal dispersion rate was characterized by using CO pulse adsorption.

**Table 3:** Fitting parameters obtained from the ethylene adsorption isotherms of Ag/NZ5(X) samples.

Adsorbent	*q_m,p_* (mg·g^−1^)	*q_m,c_* (mg·g^−1^)	*b_p_* (kPa^−1^)	*b_c_* (kPa^−1^)	R^2^
Ag/NZ5(2.0)	2.23	0.038	5.8 × 10^−3^	3.2 × 10^16^	0.99
Ag/NZ5(2.5)	3.78	0.059	4.8 × 10^−3^	1.9 × 10^21^	0.99
Ag/NZ5(3.0)	4.13	0.053	3.9 × 10^−3^	1.4 × 10^18^	0.99

## Data Availability

Data are contained within the article and Supplementary Materials.

## Supplementary Materials

The following supporting information can be downloaded at: https://www.mdpi.com/article/10.3390/molecules29050981/s1, Figure S1: experimental devices; Figure S2: Ag NPs diameter distribution of Ag/NZ5(2.5) and TEM image for the Mapping analysis; Figure S3: adsorption performance of Ag/NZ5(2.5) at different temperatures; Figure S4: reusability of Ag/NZ5(2.5); Figure S5: CO pulse adsorption profile and ethylene adsorption isotherms of the adsorbents; Figure S6: banana storage experiment of Ag/NZ5(2.5).
