# Supplementary material for: Enhanced Adsorption of Trace Ethylene on Ag/NZ5 Modified with Ammonia: Hierarchical Structure and Metal Dispersion Effects
Source: Molecules. 2024 Feb 23;29(5):981. doi: 10.3390/molecules29050981 (PMC10935363; doi:10.3390/molecules29050981)
Supplement: Supplementary file 1 [file molecules-29-00981-s001.zip › molecules-2857339-supplementary.pdf]

Enhanced Adsorption of Trace Ethylene on Ag/NZ5 Modified with Ammonia: Hierarchical Structure and Metal Dispersion Effects

Ying Qi, Huaming Yang, Chunli Li * and Hao Li *

National-Local Joint Engineering Laboratory for Energy Conservation in Chemical Process Integration and Resources Utilization, School of Chemical Engineering and Technology, Hebei University of Technology, Tianjin 300130, China; 201911501005@stu.hebut.edu.cn (Y.Q.); 202211501018@stu.hebut.edu.cn (H.Y.)

* Correspondence: lichunli_hebut@126.com (C.L.); hglh@hebut.edu.cn (H.L.)

Supporting Information Table of Contents:

1. **Figure S1:** Experimental devices for ethylene adsorption.
2. **Figure S2:** (a) Particle diameter distribution of Ag/NZ5(2.5) obtained from TEM, exhibiting an average diameter of 2.9 nm; (b) TEM image used for the elements Mapping analysis.
3. **Figure S3:** Adsorption performance of Ag/NZ5(2.5) at different temperatures, confirming an adsorption process rather than oxidation.
4. **Figure S4:** (a) Breakthrough curves of Ag/NZ5(2.5) over six consecutive cycles; (b) Adsorption capacity of Ag/NZ5(2.5) over six consecutive cycles, demonstrating the reusability of the adsorbents.
5. **Figure S5:** (a) CO pulse adsorption profile of Ag/NZ5(2.5), used for calculating the metal dispersion rate; (b) Ethylene adsorption isotherm and fitting curves of Ag/NZ5(X) adsorbents.
6. **Figure S6:** Banana storage experiment of Ag/NZ5(2.5), proving the application potential for preserving F&V.
7. Characterizations of the Ag/NZ5(X) adsorbents

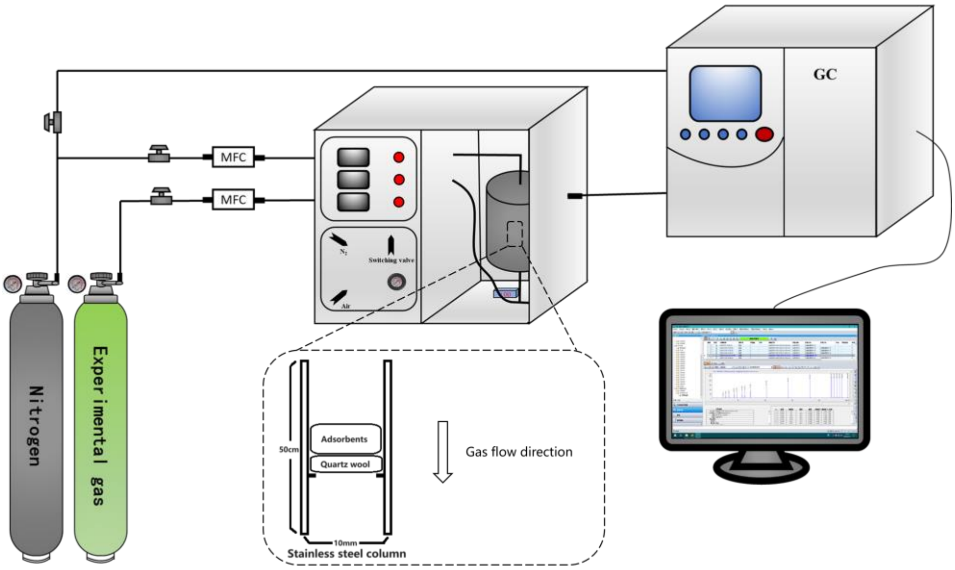

Figure S1. Experimental devices for ethylene adsorption.

For measuring the breakthrough curves, an Agilent 7820A gas chromatograph (GC) equipped with an FID detector and a HP-Plot Q column was used to detect the ethylene concentrations in the gas flow (detection limits as low as 0.1 ppm). A single layer of adsorbents supported by a thin layer of quartz wool was used to remove ethylene from the gas flow. In addition, the experiment was conducted in an indoor environment, and as the experimental gas contained no water vapor, the adsorption process can be considered unaffected by humidity.

$$R\ (\%) = (1 - \frac{C_{outlet}}{C_{inlet}}) \times 100\% \quad (1)$$

$$\mathrm{C\ (mg{\cdot}g^{-1})} = \frac{\int_0^T f(t)dt}{\mathrm{m}_{ads}} \times 85\ \mathrm{mL{\cdot}min^{-1}} \times 100\ \mathrm{ppm} \times 1.143\ \mathrm{mg{\cdot}mL^{-1}} \quad (2)$$

Where

R is the ethylene removal rate of Ag/NZ5(X); C_{inlet} and C_{outlet} are the ethylene concentrations of the inlet and outlet gas stream, respectively.

C is the adsorption capacity of Ag/NZ5(X); *T* represents the minutes of the moment that the removal rate reached zero; *f(t)* is the breakthrough curves of the adsorbents; *t* represents the minutes of the experiment duration; m_{ads} is the grams of Ag/NZ5.

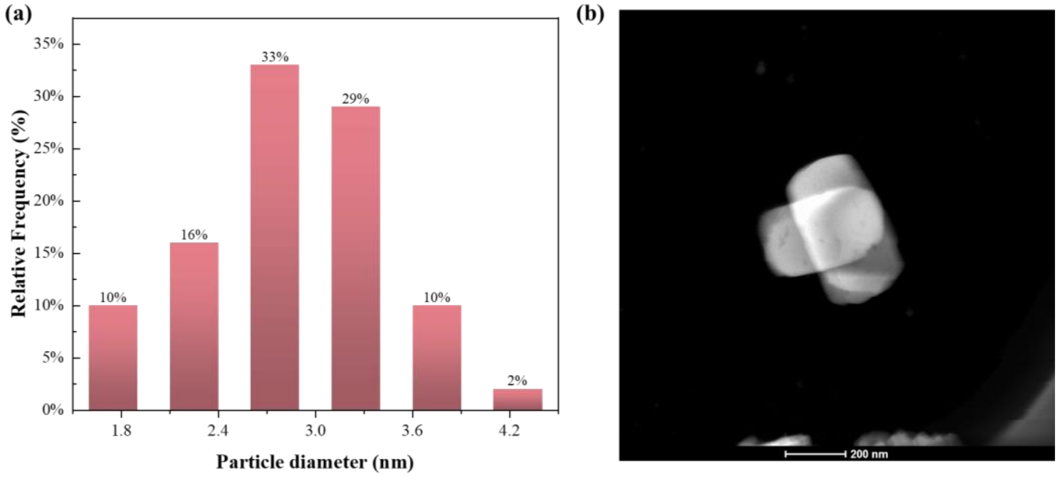

Figure S2. (a) Particle diameter distribution of Ag/NZ5(2.5) obtained from TEM, exhibiting an average diameter of 2.9 nm; (b) TEM image used for the elements Mapping analysis.

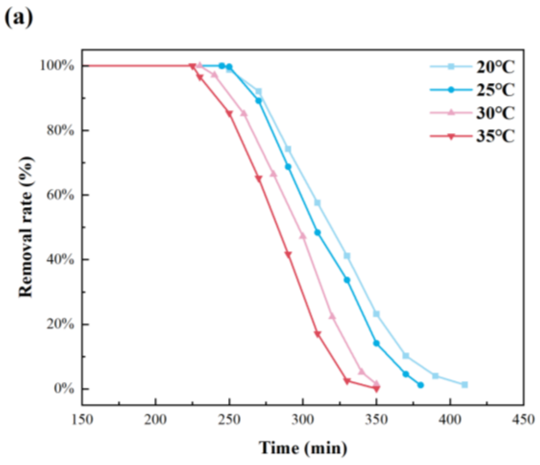

Figure S3. Adsorption performance of Ag/NZ5(2.5) at different temperatures, confirming an adsorption process rather than oxidation.

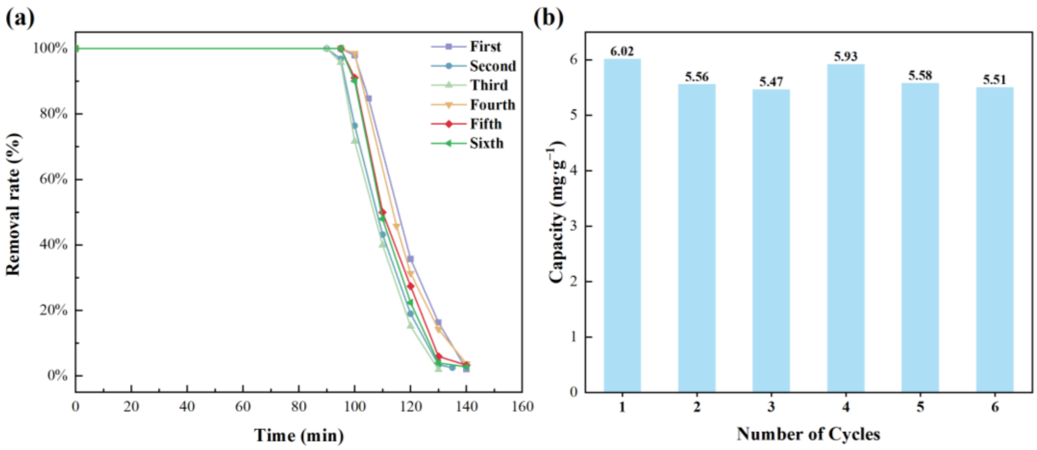

Figure S4. (a) Breakthrough curves of Ag/NZ5(2.5) over six consecutive cycles; (b) Adsorption capacity of Ag/NZ5(2.5) over six consecutive cycles, demonstrating the reusability of the adsorbents.

The used samples were also regenerated under a relatively mild condition (300 °C, 1 h) in another experiment. The results indicate that more than 80% of the capacity could still be recovered after six consecutive cycles (data not shown).

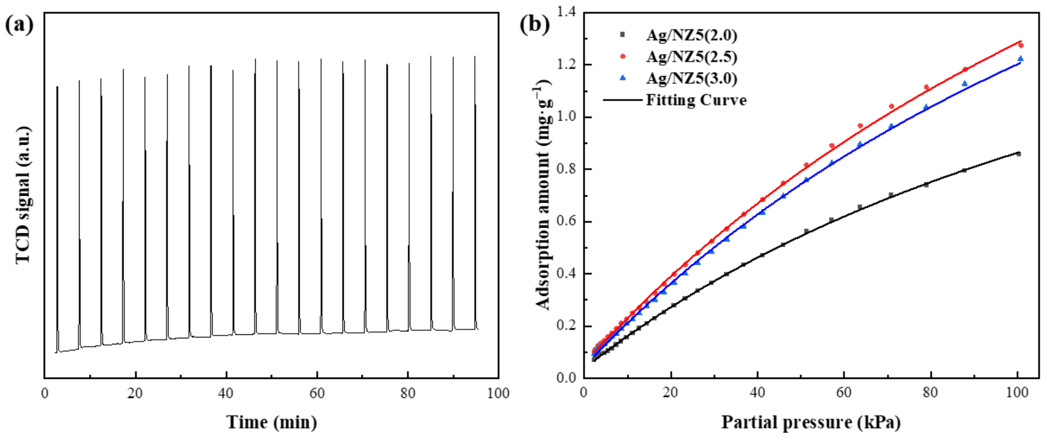

Figure S5. (a) CO pulse adsorption profile of Ag/NZ5(2.5), used for calculating the metal dispersion rate; (b) Ethylene adsorption isotherm and fitting curves of Ag/NZ5(X) adsorbents.

The value of parameter *s* in the isotherm equation was 7 for the fitting process of this work.

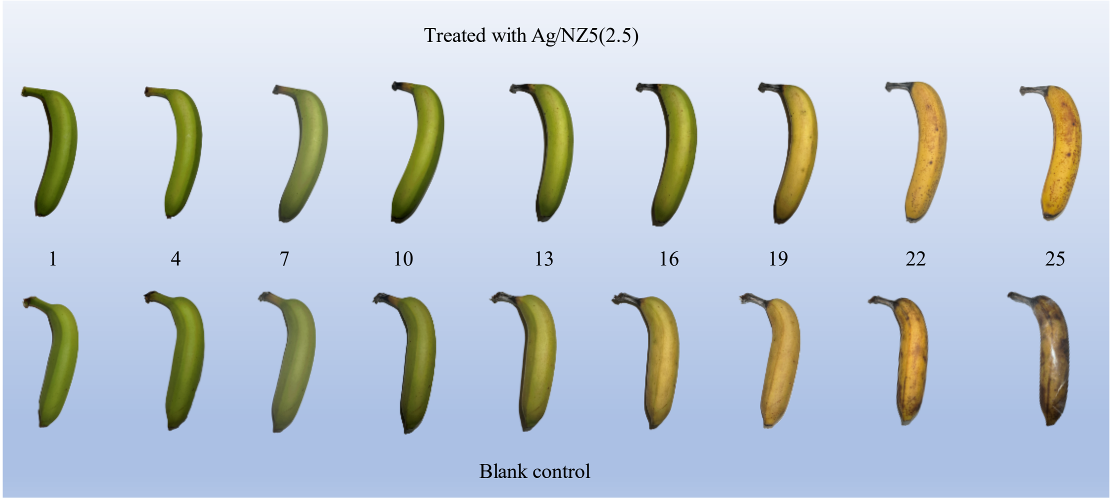

Figure S6. Banana storage experiment of Ag/NZ5(2.5), proving the application potential for preserving F&V.

Two similarly shaped and sized banana fingers were selected from the same cluster to ensure uniform initial maturity. These selected bananas were sealed in airtight bags to isolate them from external environmental factors except temperature, which might influence their ripening process. The adsorbents were placed on glass petri dishes and sealed along with the bananas in the bags, while the control group only contained empty petri dishes. The sealed bags were then placed in a constant temperature chamber set at 25 °C for the duration of the experiment. At fixed time on each day, the bananas were removed from the sealed bags and photographed before being sealed again. The actual preservation capability of the adsorbents was assessed based on the appearance of the bananas in the photographs.

Characterizations of the Ag/NZ5(X) adsorbents

The X-ray diffraction spectra (XRD) of the adsorbents were obtained by using a Bruker AXS D8 Discover X-ray diffractometer to determine the crystallinity and composition.

The structural parameters of the adsorbents were calculated by using a BSD-PS1 adsorption device, and the adsorption-desorption isotherms of N_2 were obtained at −196 °C, while the ethylene adsorption isotherms at 25 °C were also obtained by using this device.

The surface morphology and crystal structure of the adsorbents were obtained by using a Zeiss-SIGMA HD scanning electron microscope (SEM) and a JEM-2100F transmission electron microscope (TEM), while the EDS element mapping was recorded by using TEM as well.

Fourier-transform infrared spectroscopy (FT-IR) characterization was obtained on a Thermo Scientific Nicolet iS20 spectrometer.

The CO pulse adsorption experiments were conducted using a Micromeritics AutoChem II 2920 chemisorption apparatus to determine the metal dispersion rate. The sample was reduced by 10%H_2/Ar mixture gas at 200 ℃ before the CO pulse titration.

The silver-loaded contents were determined by a Shimadzu ICPE-9800 inductively coupled plasma emission spectrometer (ICP-OES).

Hydrogen temperature-programmed reduction (H_2-TPR) and temperature-programmed desorption of ethylene (C_2H_4-TPD) spectra were conducted on a PCA-

2200 chemisorption apparatus to determine the strength of metal-support interaction and the active sites of the adsorbents.
